# Disclosure of Sexual and Physical Violence: The Role of Violence Characteristics, Social Support, and Trauma-Related Shame in a Norwegian Population-Based Sample

**DOI:** 10.1177/08862605261441617

**Published:** 2026-05-07

**Authors:** Louisa Cheng Seifert, Ida Frugård Strøm, Tore Wentzel-Larsen, Anja Duun Skauge, Maria Teresa Grønning Dale

**Affiliations:** 1 Norwegian Centre for Violence and Traumatic Stress Studies, Oslo, Norway; 2 University of Oslo, Norway; 3 Center for Child and Adolescent Mental Health, Eastern and Southern Norway, Oslo, Norway

**Keywords:** help seeking reporting disclosure, child maltreatment, disclosure of domestic violence, intimate partner violence abuse, help seeking reporting/disclosure, intimate partner violence abuse, help seeking reporting disclosure, sexual assault, violence exposure

## Abstract

While disclosure of sexual and physical violence is crucial for providing support to violence-exposed individuals, many delay or abstain from disclosing to others. Research has examined various factors associated with disclosure, but often with a focus on either childhood or adulthood violence by specific perpetrators. This study utilized data from a telephone survey conducted with a Norwegian population-based sample of individuals aged 18 to 74 years (*N* = 4,299) exposed to lifetime forcible rape (*n* = 337) and severe physical violence in adulthood (*n* = 1,626). We aimed to examine associations between a wide range of violence characteristics (age at onset, fear of severe injury or death, and relationship to the perpetrator) and interpersonal factors (trauma-related shame and perceived social support), and disclosure of (a) forcible rape and (b) severe physical violence was analyzed separately. Descriptive analyses showed that 81% of individuals exposed to forcible rape and 91% of those exposed to severe physical violence had disclosed to someone. Adjusted logistic regression analyses indicated that higher levels of perceived social support, and particularly fear of severe injury or death, were significantly associated with higher odds of disclosure of both sexual (Odds ratio [OR] = 7.66, 95% confidence interval [CI] [3.49, 19.40], *p* < .001) and severe physical violence (OR = 2.32, 95% CI [1.50, 3.69], *p* < .001). A close relationship to the perpetrator and older age at onset were significantly associated with increased odds of disclosing severe physical violence, but not for forcible rape. Although differences were observed between specific violence characteristics and interpersonal factors and the disclosure of sexual and physical violence, the overarching similarities indicate the presence of shared pathways to disclosure that may enhance the well-being and recovery of individuals impacted by interpersonal violence. Broadening public perceptions of violence beyond physical, and often more “stereotypical,” violence may help foster environments where individuals feel safe to disclose.

## Introduction

Exposure to physical and sexual violence is a major public health concern linked to potentially detrimental health outcomes such as PTSD, depression, somatic disorders, and substance abuse (Campbell et al., 2009; Dworkin et al., 2017; Easton et al., 2026; Hagland et al., 2025; Kilpatrick et al., 2003). Some studies find that approximately 20% of women and 7% of men have experienced severe sexual violence, such as rape or attempted rape, during their lifetime (S. G. Smith et al., 2018), while 40% of men report exposure to severe physical violence in their adolescence or adulthood by a non-intimate partner, compared with 12% of women (Fanslow et al., 2022). Although debated in the literature, the notion of “severe” physical and sexual violence here refers to acts of violence that are legally and socially widely considered to be more offensive and/or capable of causing more serious injury. Women are more often exposed to severe physical violence by an intimate partner (Dale et al., 2023; Öberg et al., 2021). Despite the high prevalence rates, a large proportion of violence-exposed individuals delay or abstain from disclosing it to others (McElvaney, 2015; D. W. Smith et al., 2000). Given the serious consequences associated with violence exposure, identifying factors that may contribute to increased disclosure of sexual and physical violence is crucial in facilitating appropriate care and support.

The term disclosure in this article refers to the act(s) of informing someone about an assault (Campbell et al., 2015), which can be self-initiated or prompted by others and serve as functions such as seeking emotional and practical support or preventing further abuse and violence (Bussey & Grimbeek, 1995; Campbell et al., 2015). Although disclosure and help-seeking are often used interchangeably with help-seeking, disclosure does not explicitly imply that the violence-exposed individual wants or seeks help even though this might be an implicit motivation for some who disclose (Campbell et al., 2015). Given the interchangeable use in literature, this study also draws on help-seeking literature when it overlaps substantially with our broad definition of disclosure and/or when empirical findings were considered relevant for this study.

Nondisclosure or long delays of violence disclosure are associated with increased PTSD symptoms, stress, and depression compared with violence-exposed individuals who have disclosed (Ahrens et al., 2010; Easton, 2019), indicating that disclosure may create opportunities for help and support that can reduce the potential negative health impact of violence. Formal support systems can provide psychosocial interventions that can reduce negative mental health symptoms (Dworkin & Schumacher, 2018). Disclosure to informal networks may help mitigate adverse health outcomes by providing social validation and reinforcing that the individual is not to blame (Bussey & Grimbeek, 1995), while also providing practical assistance and encouragement to seek formal support (Hulley et al., 2023; Starzynski et al., 2007). However, most literature has examined formal and informal disclosure separately. Examining disclosure to both informal and formal networks is important to get a comprehensive understanding of factors that may improve overall health outcomes for violence-exposed individuals.

Studies on disclosure rates often focus on specific types of sexual and physical violence in either childhood or adulthood by specific perpetrators, but little is known about how disclosure differs across sexual and physical violence. For example, studies on adult disclosure of childhood sexual abuse (CSA) and intimate partner violence (IPV) in adulthood suggest that approximately 20% to 30% of their participants had not disclosed prior to study participation (Bottoms et al., 2016; Fanslow & Robinson, 2010; Starzynski et al., 2005). These studies are commonly conducted with narrow samples that have limited variation in sociodemographic and geographic characteristics (e.g., female college populations or help seekers). Consequently, differences in disclosure rates across such studies could be attributed to sample characteristics, limiting the generalizability. Examining disclosure of sexual and physical violence, comprising a wide range of violence characteristics, in the general population might uncover important differences that can inform future research.

A growing body of research finds that violence characteristics (e.g., the age at onset, fear of severe injury or death, and relationship to the perpetrator), and interpersonal factors (e.g., perceived social support and trauma-related shame) are significantly associated with disclosure of specific types of sexual and physical violence, such as CSA and IPV (Alaggia et al., 2019; Lelaurain et al., 2017; Starzynski et al., 2005; Sylaska & Edwards, 2014; Ullman, 2023a). This research often employs social ecological models to conceptualize influences on disclosure at individual, intrapersonal, and contextual levels, such as the *Feminist Social-Ecological Model of Sexual Assault Disclosure* by Liang et al. (2005). This model suggests that whether someone discloses or seeks help depends on whether the violence is recognized and defined as a problem, which is shaped by contextual factors such as cultural norms and stigma, and individual factors such as sociodemographic background, characteristics related to the violence exposure, and social network (Ullman, 2023c).

However, because existing literature is fragmented by specific types of violence, violence characteristics, and homogeneous samples, we have limited knowledge on how violence characteristics and interpersonal factors are associated with disclosure of sexual and physical violence in the general population. The next section summarizes the literature on selected violence characteristics and interpersonal factors that are considered important for disclosing sexual and physical violence.

### Violence Characteristics and Disclosure

Age at onset, defined as the age when first exposed to violence, has primarily been explored in research on CSA disclosure in adult samples. Findings suggest that older age at onset is associated with a higher likelihood of disclosure during adulthood (Alaggia et al., 2019; Bottoms et al., 2016; McElvaney, 2015). However, to our knowledge, this has been less explored for violence exposure in adulthood, which is likely due to convenience sampling with limited variation in age at onset. Nevertheless, studies hypothesize that older age at onset may increase the likelihood of disclosure due to greater awareness of violence, a broader social network to disclose to, and more distance from family perpetrators (Bottoms et al., 2016; D. W. Smith et al., 2000).

Fear of severe injury or death has consistently been found to an increased likelihood of disclosing sexual and physical violence in childhood and adulthood (Ansara & Hindin, 2010; Barret & St. Pierre, 2011; Bottoms et al., 2016; Fanslow & Robinson, 2010; Lelaurain et al., 2017). Fear of severe injury or death is commonly used as a measure of violence severity, although multiple operationalizations of severity exist (see Lelaurain et al., 2017) for an extensive overview). As proposed by Liang et al. (2005), increased perceptions of severity could promote disclosure or help-seeking behavior because this makes the violence more identifiable and recognizable as problematic and intolerable according to societal norms (Lelaurain et al., 2017; Liang et al., 2005; Ullman, 2023c).

Having a close relationship to the perpetrator (e.g., the perpetrator is a family member or partner) is found to decrease the likelihood of disclosure compared with having a more distant relationship to the perpetrator for both sexual and physical violence (Alaggia et al., 2019; Bottoms et al., 2016; Starzynski et al., 2005). When the perpetrator is a part of the family or household, the perpetrator is often in a better position to control and threaten over time, and the emotional attachment to the perpetrator causes more emotional distress due to a sense of loyalty and fear of disruption in the family and social environment (Alaggia et al., 2019; Bussey & Grimbeek, 1995; Lelaurain et al., 2017; Liang et al., 2005).

### Interpersonal Factors and Disclosure

High levels of trauma-related shame have consistently been found to impede disclosure of both sexual and physical violence across the lifespan (Kennedy & Prock, 2018; Lemaigre et al., 2017; McElvaney et al., 2014; Pijlman et al., 2023; Sylaska & Edwards, 2014). Shame is an emotional response in which individuals feel deeply defective or unworthy because they perceive their personal attributes or behavior as socially unacceptable or blameworthy (Gilbert, 2000; Kennedy & Prock, 2018). It is also argued to be related to avoidance behavior as an aversion to being shamed (Catton et al., 2023). Studies find that sexual violence entails more shame than other potential traumatic events such as physical violence (Amstadter & Vernon, 2008), which is hypothesized to be due to more victim-blaming messages in society related to sexual violence.

Studies suggest that higher perceived social support elicits disclosure of violence (Hulley et al., 2023; Jonzon & Lindblad, 2004; Liang et al., 2005; Ullman, 2023a). Perceived social support is argued to represent relatively stable perceptions of general availability of social support or quality of relationships, such as being more confident in receiving practical help and/or emotional support (Barrera, 1986; Kaul & Lakey, 2003). Such perceptions may imply lower anticipatory stigma, in other words, expectations of receiving fewer negative reactions from others after a potential disclosure (Kennedy & Prock, 2018; Overstreet & Quinn, 2013). Furthermore, perceptions of strong social support may suggest a social network that is more likely to facilitate disclosure directly through questions and prompts in conversations or upon suspicion that something is “wrong” (Ullman, 2023a). However, overlapping relationships between the perpetrator and the informal social network might reduce one’s willingness to disclose despite perceptions of high social support in general (Dworkin et al., 2016; Hulley et al., 2023).

## This Study

To address limitations in previous research, this study examines disclosure among individuals exposed to forcible rape and/or severe physical violence in a Norwegian population-based sample of both men and women aged 18 to 74 years. Specifically, we sought to address the following research question: What is the association between violence characteristics, more specifically, age at onset, fear of severe injury or death, relationship to the perpetrator, and interpersonal factors, more specifically, trauma-related shame and perceived social support, and disclosure of (a) forcible rape, and (b) severe physical violence?

Based on previous literature, although focused on specific violence types and more homogeneous samples, hypotheses were formulated to suggest the expected directions of relationships. For both forcible rape and severe physical violence, greater odds of disclosure were expected for those with:

**H1:** Higher age at onset
**H2:** Experienced fear of injury or death
**H3:** A less close relationship to the perpetrator
**H4:** Lower levels of trauma-related shame
**H5:** Higher levels of perceived social support

## Research Data and Methods

The current study is based on two separate subsamples of individuals who reported violence exposure to (a) forcible rape during their lifetime and (b) severe physical violence in adulthood, in the survey *The Norwegian Prevalence study on Violence and Abuse in Norway* (Dale et al., 2023). The survey data were collected between 2021 and 2022 from a random sample of individuals aged 18 to 74 years, drawn from the Norwegian Population Registry (*N* = 75,000), which includes individuals registered as permanent residents in Norway. Postal invitations were sent to all potential participants, and invitees were subsequently called by interviewers for their consent to participate. Those who could answer the survey in English or Norwegian were eligible for inclusion. The questionnaire had similar formulations in English and Norwegian, and translations were conducted by a certified translation agency.

To increase representativity, the sample was stratified by age, sex, and county. The survey interviews were primarily conducted via telephone, using Computer-Assisted Telephone Interview, following the strategy developed by Kilpatrick and colleagues (2003; Resnick et al., 1993). Please see the Supplemental Material for the flow chart of the sampling procedure and refer to Strøm et al. (2025), Skauge et al. (2025), and Dale et al. (2025) for more detailed information about the data collection.

### Participants

A total of 4,299 individuals (2,100 female, 2,195 male, 4 nonbinary respondents) consented to participate and completed the survey interview, resulting in a response rate of 26% among those reached by telephone. The sample was similar to the general adult population in Norway with regard to distribution of geographic residency and gender, but individuals with immigrant background are underrepresented, while individuals with university education and older age groups were overrepresented (Dale et al., 2023).

Among the total sample of 4,299 participants, 337 individuals (8%) reported to have been exposed to forcible rape during their lifetime, while 1,626 individuals (38%) reported to have been exposed to severe physical violence in adulthood. Respondents who answered affirmatively to questions about exposure to violence (i.e., forcible rape and/or severe physical violence) received follow-up questions about the violence incident(s) and the disclosure of the specific violence type. Due to separate follow-up questions for rape and physical violence, two separate subsamples were created for participants who reported exposure to (a) forcible rape and (b) severe physical violence. Participants who reported exposure to both types of violence (*n* = 189) are included in both subsamples to make each sample as representative of both groups as possible, since multi-victimization is common (Fanslow & Robinson, 2011). Missing on the variable measuring disclosure of either rape (missing, *n* = 2) or physical violence (missing, *n* = 16) resulted in exclusion from each analysis.

Considering the sensitive topic of the survey and the potentially vulnerable situation of the participants, multiple efforts were made to reduce potential negative consequences of participating in the survey. Interviewers were instructed to ask whether the participant could answer questions in private, to reduce the risk of participants being overheard by others during the telephone interview. Participants had the option to reschedule the interview if these conditions were not met. Questions and answer options related to violence exposure were primarily possible to answer with a yes/no, which could further reduce the risk of sensitive information being overheard by others. At the end of the interview, participants were offered mental health services if needed. To reduce misinterpretations and stigma related to violence terms such as “rape,” behavioral-specific questions were used (Harned, 2004). The study was funded by the Norwegian Ministry of Justice and Public Security and other Norwegian ministries and approved by the Regional Committee for Medical and Health Research Ethics.

## Measures

### Violence Exposure

*Exposure to severe physical violence* was measured with the question: “Have you ever—after turning 18—experienced someone physically attacking you in the following ways: (a) hit you with their fist or a hard object, (b) kicked you, (c) strangled you, (d) beat you up, (e) threatened you with a weapon, (f) physically attacked you in some other way” (yes/no). Respondents with affirmative answers to one or more items were coded as exposed to severe physical violence. A modified version of the Conflict Tactics Scale (Straus, 1990) was used. Items with the largest potential of causing harm were selected to capture physical violence with a higher criminal sanction according to Norwegian law.

*Exposure to forcible rape* was measured with the following questions: “Has anyone ever made you have (a) sexual intercourse, (b) oral sex, (c) anal sex, or (d) put their fingers or objects inside their vagina or anus against your will by using physical force or threatening to harm you or someone close to you” (yes/no). Respondents with exposure to one or more items were coded as exposed to forcible rape. This set of questions was first introduced in the National Women’s Study (Kilpatrick et al., 1992) and corresponds with the legal definition of rape in Norwegian law.

### Outcome Variables: Disclosure of Violence Exposure

The separate measure of disclosure of severe physical violence and forcible rape comprised two questions: (a) “Have you ever talked to health care professionals about this/these incidents? and (b) “Have you told anyone [else] about this/these incidents? Options provided for these two questions were as follows: (a) “yes” (if one incidents/no), (b) “yes, about everything” (if more than one incident), (c) “yes, about some of them” (if more than one incident), (d) “no.” Respondents who responded affirmatively “yes” or “yes, about everything” or “yes, about some of them” to at least one of the disclosure questions were categorized as having disclosed severe physical violence or forcible rape. Respondents were coded as missing on disclosure if they either had missing on both follow-up questions on disclosure (healthcare professionals or others), or if they had missing on one question in combination with “no” on the other question. Moreover, respondents who were exposed to forcible rape or severe physical violence but did not answer a filter question on whether the violence exposure(s) happened once or multiple times, did not receive follow-up questions on the violence exposure and were coded as missing.

### Violence characteristics

#### Age at Onset

Respondents who reported exposure to forcible rape or severe physical violence were asked: “about how old were you when it happened?” Those who reported having been exposed to multiple types of forcible rape were asked: “how old were you the first time it happened?,” while those exposed to multiple items of severe physical violence, were asked to report the age at onset for each of the items one had answered affirmatively to. For those who answered affirmatively to multiple items, the lowest age reported across all items was used to measure age at onset. Missing was coded for those who did not report age at first exposure to forcible rape(s) or physical violence, and for those who did not answer the filter question and did not receive any questions about age at onset.

#### Fear of Severe Injury or Death

This was measured with the following question for both severe physical violence and forcible rape separately: “Were you ever afraid that you were going to be seriously injured or killed?” (yes/no).

#### Relationship to the Perpetrator

A hierarchical variable was constructed because it was possible to have multiple perpetrators, based on answers to the following question: “What was your relationship to the person(s) who did this?” Three mutually exclusive categories were created based on the hypothesized “closeness” to the perpetrator: (a) *Close perpetrator*, (b*) Other known perpetrator*, and (c) S*tranger. Close perpetrator* comprised partner/former partner (former or current spouse, cohabitant, partner girlfriend/boyfriend), parents/caregivers (biological parents, stepparents, mother’s or father’s partner), other family members (brother/stepbrother, sister/stepsister, grandparents, children/stepchildren, other adult relatives, other relatives who are children). *Other known perpetrator* comprised friends and acquaintances, both children and adults (neighbor, colleague, customer/client/patient, adult leader of a youth activity, coach, teacher/other school staff, doctor/ psychologist/healthcare professional, religious leader, social worker, supervisor, pupils, other known children/adolescents/adults). *Stranger* comprised unknown adults/children.

Respondents were assigned the category based on the closest relationship to a perpetrator reported. Consequently, if a respondent reported at least one of the abusers to be a former/current partner, it was coded as a “close” relationship to the perpetrator, regardless of also reporting other perpetrators in the less close categories of “other known perpetrators” or “stranger.” Missing was assigned to respondents who did not answer affirmatively to any of the perpetrator items, and those who did not receive questions about perpetrators due to missing on the filter question.

### Interpersonal Factors: Perceived Social Support and Trauma-related Shame

#### Perceived Social Support

Measured by the mean score of the following four items from the Crisis Support Scale (Joseph et al., 1992): a) When you feel the need to talk, how often is someone willing to listen to you? b) Are you able to talk about your thoughts and feelings? c) Do people show you sympathy and support? d) Is there someone who can give you practical help? This set of questions was measured on a 5-point Likert-scale ranging from (0) “never,” (1) “rarely,” (2) “sometimes,” (3) “often or very often,” and (4) “always.” The mean score was calculated from respondents who answered at least two items. All respondents received these questions independent of exposure to violence. Cronbach’s alpha was .82.

#### Trauma-Related Shame

All respondents who reported exposure to one or more potentially traumatic situations in the survey (including forcible rape and severe physical violence) were asked the following questions: (a) Have you been worried about what people might think of you after what happened? (b) Have you tried to conceal what happened, or any part of it? (c) Have you felt ashamed about any part of what happened? (d) Have you looked down on yourself after what happened? These items could be scored on a scale from 0 to 2 (0 = “no,” 1 = “yes, a little,” 2 = “yes often”). A mean score was computed for respondents with answers for at least two items and used as a continuous variable in subsequent analyses. Cronbach’s alpha was .81. The scale used in this study was a shortened version of the Shame and Guilt After Trauma Scale in Aakvaag et al. (2016). Since respondents could be exposed to multiple types of potentially traumatic life events, this measure of shame might not be directly associated with the specific form of violence analyzed in this study, but trauma-related shame more generally.

#### Sociodemographic Variables

*Age* was measured as a continuous variable. Gender comprised “female,” “male,” and “non-binary.” No participants identifying as “non-binary” reported exposure to either severe physical violence or forcible rape. *Immigrant background* was defined as respondents who were born outside of Norway with two parents who were also born outside of Norway. *Perceived family financial situation* was measured with the question: “How well off do you think your family is compared to most people?” with the following response options (a) “Better off,” (b) “About the same as most people,” (c) “Worse off.” *Level of education* was measured with the question: “What is your highest completed education?” with the following responses: “primary school or less,” “high school,” and “university.”

### Statistical Analysis

First, we conducted unadjusted logistic regression models for both subsamples to examine how each of the sociodemographic variables, violence-related characteristics, and interpersonal factors were associated with disclosure of forcible rape and severe physical violence. Subsequently, multiple logistic regression analyses for each of the subsamples were applied to examine the relationship between each violence-related characteristic, and interpersonal factor, and disclosure, adjusting for the sociodemographic variables: *age, gender, education level, perceived family financial situation*, and *immigrant background*.

For the subsample with individuals exposed to severe physical violence, a fully adjusted model was estimated comprising all violence-related characteristics, interpersonal factors, and sociodemographic background variables. It was not possible to include a fully adjusted model for the subsample on forcible rape due to low sample size. Lastly, bootstrapping was performed to compare the estimates from the adjusted logistic models across the two subsamples for the estimates whose confidence intervals partly overlapped. This approach aimed to examine whether differences in odds ratio estimates between the subsamples were likely to be attributable to random sampling variation or indicative of underlying differences between the subsamples. Bootstrapping facilitates this examination by allowing us to compute confidence intervals for odds ratios for each subsample and their difference, and is suitable for overlapping samples.

To ensure the assumptions of logistic regression were met, we checked the absence of substantial multicollinearity and the linearity of the logit for the continuous variables, *age*, and *age at onset*. Multicollinearity was checked by variance inflation factor (VIF). VIF scores were lower than VIF ≥ 10, commonly regarded as substantial multicollinearity (Cohen et al., 2022). Linearity of logit was checked for the continuous variables by comparing the results of the ordinary logistic regression model and logistic regression model incorporating restricted cubic splines. This evaluation was conducted using the Anova function to test for significant differences between the two models. The analysis did not indicate violations of the assumption of absence of linearity of logit.

All statistical analyses were conducted in R version 4.2.3, with the package boot (Canty & Ripley, 2025) for bootstrapping and rms (Harrell & Frank, 2023) for restricted cubic splines.

## Results

Tables 1 and 2 show that 271 out of 335 individuals (81%) who were exposed to forcible rape disclosed the incident(s) while 1,468 out of 1,614 individuals (91%) exposed to severe physical violence reported having disclosed their experiences. Differences between the two subsamples with regard to sociodemographic characteristics, violence-related characteristics, and interpersonal factors could also be observed. As shown in Table 1, individuals who were exposed to forcible rape mostly consisted of females (89%) and the mean age of the respondents was 45 years (standard deviation [*SD*] = 15). The mean age of onset for forcible rape was 18 years (*SD* = 8), and 41% reported having a close relationship to at least one perpetrator.

**Table 1.** Overall Frequency Distribution of Sociodemographic Characteristics, Violence Characteristics, and Interpersonal Factors by Disclosure Status among Individuals Exposed to Forcible Rape.

Variables	Total sample frequency *n* (%)/Mean (*SD*)^a^	Distribution by dependent variable *n* (%) /Mean (*SD*)^a^		
	Overall *n* = 335 (100%)	Nondisclosure *n* = 64 (19%)	Disclosure *n* = 271 (81%)	*p*-value^b^
Sociodemographic characteristics				
Gender				.467
Male	36 (11)	9 (25)	27 (75)	
Female	299 (89)	55 (18)	244 (82)	
Age	44.7 (15.4)	48.3 (14.6)	43.8 (15.5)	**.029**
Age categorical^c^				—
18–29	78 (23)	10 (13)	68 (87)	
30–39	62 (19)	10 (16)	52 (84)	
40–49	64 (19)	10 (16)	54 (84)	
50–59	56 (17)	17 (30)	39 (70)	
60–69	57 (17)	13 (23)	44 (77)	
70–74	18 (5)	4 (22)	14 (78)	
Perceived family financial situation				.529
Better off than most people	68 (21)	11 (16)	57 (84)	
Same as most people	181 (55)	39 (22)	142 (78)	
Worse off than most people	82 (25)	14 (17)	68 (83)	
Education level				.767
University level	189 (57)	38 (20)	151 (80)	
High school	119 (36)	20 (17)	99 (83)	
Primary school or less	26 (8)	5 (19)	21 (81)	
Immigrant background				.555
No immigrant background	315 (94)	59 (19)	256 (81)	
Immigrant background	20 (6)	5 (25)	15 (75)	
Violence characteristics				
Age at onset	18.1 (7.5)	19.9 (8.2)	17.7 (7.3)	.050
Fear of severe injury/death				**<.001**
No	199 (60)	55 (28)	144 (72)	
Yes	132 (40)	7 (5)	125 (95)	
Relationship to the perpetrator (hierarchical)				.278
Stranger	72 (22)	9 (12)	63 (88)	
Known	121 (37)	26 (21)	95 (79)	
Close	135 (41)	27 (20)	108 (80)	
Interpersonal factors				
Trauma-related shame	1.2 (0.7)	1.1 (0.7)	1.2 (0.7)	.323
Perceived social support	2.9 (0.8)	2.5 (0.9)	3.0 (0.8)	**<.001**

*Note. n* = 335. SD = standard deviation. Bold values indicate statistically significant associations at *p* < .05.

a *n* (%) is reported for categorical variables; mean (*SD*) is reported for continuous variables

b *d* Asymptotic Pearson’s chi-squared tests were performed for categorical variables with *n* > 5 for all combinations. If not, exact chi-squared tests were used. Welch two-sample t-tests were performed for continuous variables.

c Only showed for illustrative purposes, but not included in subsequent analyses.

**Table 2.** Overall Frequency Distribution of Sociodemographic Characteristics, Violence Characteristics, and Interpersonal Factors by Disclosure Status Among Individuals Exposed to Severe Physical Violence.

Variables	Total sample frequency *n* (%)/Mean (*SD*)^a^	Distribution by dependent variable *n* (%)/Mean (*SD*)^a^		
	Overall *n* = 1,614 (100%)	Nondisclosure *n* = 146 (9%)	Disclosure *n* = 1,468 (91%)	*p*-value^b^
Sociodemographic characteristics				
Gender				**<.001**
Male	999 (62)	110 (11)	889 (89)	
Female	615 (38)	36 (6)	579 (94)	
Age	47.2 (14.3)	52.9 (14.0)	46.6 (14.2)	**<.001**
Age categorical^c^				—
18–29	230 (14)	13 (6)	217 (94)	
30–39	291 (18)	14 (5)	277 (95)	
40–49	361 (22)	23 (6)	338 (94)	
50–59	365 (23)	42 (12)	323 (88)	
60–69	272 (17)	40 (15)	232 (85)	
70–74	95 (6)	14 (15)	81 (85)	
Perceived family financial situation				.710
Better off than most people	514 (32)	48 (9)	466 (91)	
Like most people	874 (55)	78 (9)	796 (91)	
Worse off than most people	215 (13)	16 (7)	199 (93)	
Education level				.248
University level	951 (59)	77 (8)	874 (92)	
High school	574 (36)	61 (11)	513 (89)	
Primary school or less	85 (5)	8 (9)	77 (91)	
Immigrant background				.906
No immigrant background	1,556 (96)	140 (9)	1,416 (91)	
Immigrant background	58 (4)	6 (10)	52 (90)	
Violence characteristics				
Age at onset	22.6 (9.3)	21.1 (7.9)	22.8 (9.4)	**.021**
Fear of severe injury/death				**<.001**
No	1,032 (64)	118 (11)	914 (89)	
Yes	576 (36)	28 (5)	548 (95)	
Relationship to perpetrator (hierarchical)				.487
Stranger	701 (44)	58 (8)	643 (92)	
Known	545 (34)	50 (9)	495 (91)	
Close	362 (23)	38 (10)	324 (90)	
Interpersonal factors				
Trauma-related shame	0.4 (0.6)	0.4 (0.6)	0.4 (0.6)	.429
Perceived social support	3.2 (0.7)	2.9 (0.9)	3.2 (0.7)	**.002**

*Note. n* = 1,614. SD = Standard deviation. Bold values indicate statistically significant associations at *p* < .05.

a *n* (%) is reported for categorical variables; mean (*SD*) is reported for continuous variables

b Pearson’s chi-squared tests were performed for categorical variables; Welch’s two-sample t-tests were performed for continuous variables.

c Only showed for illustrative purposes, but not included in subsequent analyses.

In contrast, as shown in Table 2, males constituted more than half of the subsample (62%) who reported exposure to severe physical violence. The mean age was 47 years (*SD* = 14), and the average age at onset for severe physical violence exposure was 23 (*SD* = 9). Moreover, 44% of the individuals exposed to severe physical violence had been exposed to physical violence by stranger(s) exclusively, while 23% reported a close relationship to at least one perpetrator (family member or previous/present partner). Trauma-related shame levels were lower than in the subsample exposed to rape but higher than in a constructed comparison group consisting of individuals exposed to potentially traumatic events excluding sexual and physical violence (see Supplementary Material). in both subsamples, about 40% reported having been scared to get a severe injury or die during the exposure.

## Disclosure of Forcible Rape: Associations with Violence Characteristics and Interpersonal Factors

As illustrated in Table 3, unadjusted analyses showed that the violence characteristics *Age at onset* and *Fear of severe injury or death* were significantly associated with disclosure of forcible rape. In other words, individuals who reported that they experienced fear of severe injury/death at least once during the violence had a strong increase in the odds of having disclosed (unadjusted OR = 6.82, *p* < .001) compared with those who did not report having this fear. Furthermore, individuals who were younger when they were first exposed to forcible rape had an increased odds of having disclosed compared with those who were older at the time of the initial exposure. Among the interpersonal factors, social support was also significantly associated with increased odds of disclosure: Those with a higher level of perceived social support (on the scale ranging from 0 to 4) had two times higher odds of having disclosed to someone than those with a lower mean level of perceived social support.

**Table 3.** Unadjusted and Adjusted Odds Ratios for Associations between Disclosure of Forcible Rape, Violence Characteristics, and Interpersonal Factors.

Predictors	Unadjusted models			Models adjusted for sociodemographic characteristics^a^		
	OR^b^	95% CI^b^	*p*-value	aOR^b^	95% CI^b^	*p*-value
Violence characteristics						
Age at onset (OR per 5-y)	0.84	0.71, 0.99	**.038**	0.88	0.73, 1.05	.160
Fear of severe injury/death			**<.001**			**<.001**
No^c^	—	—		—	—	
Yes	6.82	3.19, 16.90		7.66	3.49, 19.40	
Relationship to perpetrator (hierarchical)			.253			.199
Stranger^c^	—	—		—	—	
Known	0.52	0.22, 1.15		0.47	0.19, 1.08	
Close	0.57	0.24, 1.25		0.55	0.22, 1.25	
Interpersonal factors						
Trauma-related shame	1.22	0.82, 1.80	.319	1.16	0.77, 1.75	.474
Perceived social support	1.99	1.44, 2.77	**<.001**	2.09	1.47, 3.02	**<.001**

Notes. Bold values indicate statistically significant associations at p < .05. Missing (NA = Not Applicable): *Age at onset* (unadjusted model: NA = 8, adjusted model: NA = 12), *Fear of severe injury/death* (unadjusted model: NA = 4, adjusted model: 9), *Relationship to the perpetrator* (unadjusted model: NA = 7, adjusted model: NA = 12), *Trauma-related shame* (unadjusted model: NA = 0, adjusted model: NA = 5), *Perceived social support* (unadjusted model: NA = 0, adjusted model: NA = 5).

a Sociodemographic characteristics adjusted for the following: gender, age, financial situation, education level, immigrant background.

b OR = Odds ratio for disclosure of forcible rape (yes); CI = Confidence Interval (lower, upper); aOR = Adjusted odds ratio.

c Reference category.

After adjusting for sociodemographic characteristics, the odds of disclosure increased further for those who reported fear of severe injury or death during the incident(s) compared with those who did not report this (adjusted odds ratio [aOR] = 7.66, *p* < .001). Additionally, the odds of disclosure also slightly increased for social support after adjustment. However, the significant negative association between age at onset and disclosure was no longer significant after adjustment for sociodemographic characteristics.

## Disclosure of Severe Physical Violence: Associations with Violence Characteristics and Interpersonal Factors

As shown in Table 4, the unadjusted analyses of the association between disclosure and interpersonal violence, individuals who were older at the onset of exposure to physical violence had increased odds of having disclosed compared with those who were younger at the age of onset. Moreover, individuals who reported fear of severe injury or death also had higher odds of having disclosed than those who did not report this fear. For the interpersonal factors, a unit increase in perceived social support (scale ranging from 0 to 4) was associated with higher odds of disclosure.

**Table 4.** Unadjusted and Adjusted Odds Ratios for Associations Between Disclosure of Severe Physical Violence, Violence Characteristics, and Interpersonal Factors.

Predictors	Unadjusted models			Models adjusted for sociodemographic characteristics^a^			Fully adjusted models^b^		
	OR^c^	95% CI^c^	*p*-value	aOR^c^	95% CI^c^	*p*-value	aOR^c^	95% CI^c^	*p*-value
Violence characteristics									
Age at onset (OR per 5-y)	1.12	1.01, 1.25	**.035**	1.13	1.02, 1.27	**.025**	1.13	1.01, 1.28	**.028**
Fear of severe injury/death			**<.001**			**<.001**			**<.001**
No^d^	—	—		—	—		—	—	
Yes	2.53	1.68, 3.94		2.32	1.50, 3.69		3.13	1.93, 5.24	
Relationship to perpetrator (hierarchical)			.493			**.005**			**.010**
Stranger^d^	—	—		—	—		—	—	
Known	0.89	0.60, 1.33		0.72	0.48, 1.10		0.73	0.48, 1.13	
Close	0.77	0.50, 1.19		0.41	0.25, 0.70		0.41	0.23, 0.73	
Interpersonal factors									
Trauma-related shame	1.12	0.84, 1.51	.446	0.78	0.56, 1.11	.167	0.93	0.63, 1.39	.715
Perceived social support	1.51	1.21, 1.86	**<.001**	1.51	1.19, 1.90	**<.001**	1.44	1.12, 1.85	**.005**

**Notes.:** Bold values indicate statistically significant associations at *p* < .05. Missing (NA = Not Applicable): *Age at onset* (unadjusted model: NA = 25, adjusted model: NA = 40), *Fear of severe injury/death* (unadjusted model: NA = 6, adjusted model: NA = 19), *Relationship to the perpetrator* (unadjusted model: NA = 6, adjusted model: NA = 21), *Trauma-related shame* (unadjusted model: NA = 2, adjusted: NA = 17), *Perceived social support*: (unadjusted model: NA = 2, adjusted model: NA = 17). Fully adjusted models: NA = 53.

a Sociodemographic characteristics adjusted for the following: age, gender, education level, perceived relative financial status, immigrant background.

b Adjusted for sociodemographic characteristics, violence characteristics, and interpersonal factors.

c OR = Odds ratio for disclosure of severe physical violence (yes), CI = Confidence Interval (lower, upper); aOR = Adjusted odds ratio.

d Reference group.

After adjusting for sociodemographic characteristics, the estimated associations between disclosure and *Age at onset* only changed slightly: while the odds of disclosure somewhat increased for individuals who were older when being exposed to severe physical violence the first time. Moreover, the odds of disclosure also slightly decreased after adjusting for sociodemographic characteristics for individuals who experienced fear of severe injury or death and disclosure. However, individuals who reported being in a close relationship to at least one perpetrator had a significant decrease in odds of having disclosed (aOR = 0.41, *p* = .005) compared with those who had perpetrator(s) who were exclusively strangers, after adjusting for sociodemographic characteristics.

When all variables were incorporated in a fully adjusted model, the odds ratio for disclosure of severe physical violence showed minimal change in relation to the various violence characteristics and interpersonal factors. However, the odds of disclosure increased for individuals who reported to have been scared to die or get severely injured during at least one violence incident after adjusting for the remaining variables (OR = 2.31, aOR = 3.13).

Finally, results from applying bootstrap resampling to compare the two subsamples (i.e. forcible rape and severe physical violence) suggested that the association between relationship to the perpetrator and disclosure for forcible rape versus severe physical violence were significantly different in the models adjusted for sociodemographic characteristics. However, there was no strong evidence that there were statistical differences between estimates for the association to disclosure for shame or social support across the two subsamples. Thus, differences in the OR estimates for relationship to perpetrator are likely attributable to underlying differences between the two groups, whereas the differences in OR estimates for social support and shame may be due to random sampling variability. Results can be viewed in the supplementary material.

## Discussion

This study sought to address previous research gaps by examining a wide range of violence characteristics and interpersonal factors in relation to disclosure of forcible rape and severe physical violence in a population-based sample.

Fear of severe injury or death was the only violence characteristic significantly associated with higher odds of disclosure of both forcible rape and severe physical violence. This finding is consistent with previous literature and suggests that violence of a more physically threatening form is recognized as problematic and unlikely to remit without help from others, which promotes disclosure (Goodman et al., 2003; Liang et al., 2005; Ullman, 2023b). This association was particularly strong for individuals exposed to forcible rape, with odds of disclosure nearly eight times higher than for those who did not report this fear. This may be attributed to societal stigma surrounding sexual violence and rape myths (persistent and false beliefs about sexual violence shaped by sexism and other discriminatory attitudes that serve to justify sexual aggression commonly against women (Lonsway & Fitzgerald, 1994)), along with social perceptions that violence with visible signs of physical injury is considered more “legitimate” (Ullman, 2023b). In contrast, the absence of visible physical injury might increase fear of negative reactions from others, such as not being believed or being blamed. Thus, disclosure of sexual violence might be particularly reliant on fear of severe injury or death because otherwise the violence might not be considered as distressing or “serious enough” to disclose (Skauge et al., 2025). It could also indicate that the violence was perceived as so traumatic that disclosure was necessary to receive medical or psychosocial help (Goodman et al., 2003).

Consistent with previous research, social support was positively associated with disclosure of both sexual and physical violence, suggesting that higher perceived social support increases disclosure (Hulley et al., 2023; Jonzon & Lindblad, 2004; Liang et al., 2005; Ullman, 2023a). Perceived social support may reduce the feelings of isolation after violence, suggesting a safer social environment for disclosing stigmatized experiences (Liang et al., 2005). The association between perceptions of higher social support and disclosure was strong among those exposed to forcible rape. Sexual violence is argued to be highly stigmatized in society, with increased levels of self-blame and anticipatory stigma commonly attributed to rape myths (Ullman, 2023b). Thus, perceptions of higher social support might be particularly important to counteract the strong societal stigma related to sexual violence, which, in turn, might enhance disclosure.

A closer relationship to the perpetrator was associated with decreased odds of disclosure among survivors of severe physical violence in adulthood. Although there is limited comparability across studies that have focused on violence by different perpetrators, a close relationship to the perpetrator is commonly argued to reduce the odds of disclosure later in life due to strong emotional attachment, fear of family disruption, and being exposed to control and threats by the perpetrator over time (Alaggia et al., 2019; Bussey & Grimbeek, 1995; Lelaurain et al., 2017; Liang et al., 2005). This association was not found for forcible rape, possibly due to underlying differences between the subsamples as implied by the bootstrap analyses.

Moreover, being older at the first time of exposure to severe physical violence was positively associated with disclosure in this study. Although physical violence was measured in adulthood, the first time experiencing this type of violence could have occurred before turning 18 years old. Previous studies on childhood violence support this finding suggesting that older age at first exposure is related to having a less close relationship to the perpetrator and a larger social network to disclose to, which potentially increase odds of disclosure later in life (Bottoms et al., 2016). However, this association was not observed among individuals exposed to forcible rape after adjusting for sociodemographic characteristics. This may reflect that they had less time to disclose, as those exposed to forcible rape on average were both younger when they participated in the study and when they were first exposed to the violence, compared with the subsample exposed to physical violence. Nevertheless, this remains unclear as we do not have data on the time elapsed between first exposure to first disclosure.

We found no significant association between shame and disclosure of sexual or physical violence, which is in contrast to prior studies on trauma-related shame and disclosure of specific types of sexual and physical violence (see Pijlman et al., 2023). Since the measure of trauma-related shame is not exclusively related to a specific violence exposure, it could reflect other traumatic events among those with multiple traumas. Moreover, shame levels might have increased after disclosure due to perceived invalidating reactions from the recipient (Catton et al., 2023), making it difficult to identify any correlation.

Lastly, our findings suggest that disclosure rates of sexual and physical violence differ. While 91% of individuals exposed to severe physical violence in adulthood reported having disclosed to someone (healthcare professional or others), this was only the case for 81% of those exposed to forcible rape during the lifetime. Still, these rates are relatively high compared with other studies of disclosure of more specific forms of both sexual violence and severe physical violence, such as Fanslow and Robinson (2010), Pijlman et al. (2023), and Bottoms et al. (2016). This could be due to our study examining violence across the lifespan with a wider range of characteristics (e.g., not limited to a specific perpetrator), and that our measures of violence exposure encompass more physically abusive violence, which has been found to increase likelihood of disclosure (Lelaurain et al., 2017).

Moreover, our disclosure rates may reflect the Norwegian context. As suggested by Liang et al. (2005), Ullman (2023b), and Kennedy and Prock (2018), societal norms and structures, such as gender equality and rape myths, are assumed to influence what individuals perceive and label as “legitimate violence,” which in turn can influence the disclosure processes. Although norms and structures may also vary within countries, results from our population-based samples could be less generalizable to contexts where the more general societal norms and structures differ greatly.

### Strengths and Weaknesses

Unlike most studies examining violence limited to certain characteristics within convenience samples, our population-based study allowed us to examine how multiple violence characteristics and interpersonal factors were associated with disclosure of forcible rape and severe physical violence within the same study, while controlling for relevant sociodemographic characteristics. While the exposure to forcible rape is measured over a lifetime and severe physical violence is only measured in adulthood, the primary goal was to examine these associations within each violence type. However, disclosure rates and differences in estimates may reflect these differences in exposure time.

Moreover, the cross-sectional design only allows us to establish associations and not causal relationships. We assume that violence characteristics and interpersonal factors influence disclosure, but there is a risk of posttreatment bias because questions related to the violence exposure are retrospective while questions about sociodemographic characteristics, social support, and shame are answered at the time of the survey. Disclosure in the past may have influenced the variables measured when participating in the study. For example, current sociodemographic characteristics may not accurately reflect the individual’s sociodemographic characteristics at the time of violence exposure or disclosure. Moreover, disclosing to someone may increase the perception of social support if the experience with disclosure is perceived as positive. Nevertheless, the literature suggests that perceived social support reflects more general availability of social support and may thus be less subject to change over time in response to specific experiences.

Our study design and dichotomous measure of disclosure prevented us from linking violence characteristics directly to separate violence episodes for individuals exposed to multiple violence incidents. This increased the risk of spurious relationships, as some violence characteristics and interpersonal factors may not reflect the content of the disclosed incident. Conversely, collecting more detailed information to separate violence episodes would have entailed a more lengthy and complex survey design that could have increased the risk of dropout for participants exposed to multiple violence episodes. Additionally, the current study did not collect information about the timing of disclosure, which could reveal important temporal aspects of how our predictors relate to delay of disclosure, as well as mental health outcomes. Thus, while our current study of disclosure contributes to expanding our understanding of how various characteristics and interpersonal factors are associated with disclosure, it remains a limited representation of a complex process that should be further explored.

Lastly, we did not have sufficient degrees of freedom to include variables in the same model for individuals exposed to forcible rape. Consequently, the observed statistical associations between disclosure of forcible rape and each violence characteristic and interpersonal factor could be attributable to remaining variables that were not included. The sample size might also be insufficient to detect statistical associations. For example, contrary to existing literature, we found no significant association between relationship to the perpetrator and disclosure of forcible rape, potentially indicating a lack of statistical power.

### Implications for Research and Policymakers

One of the main findings was the strong association between fear of severe injury or death and disclosure, which highlights the role of perceived physical danger, particularly for forcible rape. Individuals not perceiving their violence exposure as life-threatening had lower odds of disclosure, despite being exposed to severe violence. This is alarming, as they might be particularly at risk of remaining silent, reducing their opportunities to receive support and help. Raising awareness about violence beyond the most physically threating or forceful forms could make it easier for individuals to recognize and understand their own experiences as violence exposure. Moreover, psychoeducation about common psychological responses after violence could reduce self-blame and shame among individuals exposed to violence, which may in turn enhance disclosure of violence (Knipschild et al., 2024).

The second main finding was that increased levels of perceived social support were positively associated with disclosure of both sexual and physical violence, underscoring the importance of emotionally safe environments. Future efforts should ensure that violence-exposed individuals have access to low-threshold services, such as anonymous helplines and support groups, which can provide support regardless of individuals’ informal support network. Societal stigma related to violence may be subconsciously internalized and perpetuated in communities, institutions, and informal networks, which might increase fear of negative reactions from others upon disclosure (Kennedy & Prock, 2018). Thus, further studies should explore how public health interventions and policy strategies can reduce stigma surrounding interpersonal violence, including reducing victim blaming in public discourse and stigma that may be imposed by formal services, such as the police. Reducing the societal stigma of interpersonal violence may increase individuals’ perceptions of social support and create safer social environments for individuals to disclose.

Research should also examine what may enhance disclosure of a broader range of violence types throughout the lifespan that might be particularly challenging for individuals to label as violence due to widespread narratives in society about who constitutes a “legitimate victim” and messages of blameworthiness attributed to not “doing enough to stop the violence” (Ullman, 2023b). For example, online sexual abuse where individuals might have been exposed to extensive manipulation to engage in sexual activities, or unwanted sexual activity characterized by subtle coercive tactics in which individuals are led to believe it was consensual (Gemara et al., 2025).

Future studies should also include more time-sensitive measures of disclosure to better understand how violence characteristics and interpersonal factors relate to delay in disclosure. Moreover, violence-related shame, specifically, should be examined as a mediator between disclosure and various sociodemographic and violence characteristics. Lastly, including measures of multi-victimization could allow for a more nuanced understanding of how cumulative experiences of violence relate to disclosure behaviors that reflect the totality of violence-exposed individuals’ lived experiences.

## Conclusion

Our findings highlight that having experienced fear of injury or death is important for disclosure, particularly among individuals exposed to forcible rape. This indicates that violence may go undetected unless perceived as life-threatening, delaying access to support and help. Broadening public perceptions and recognizing violence beyond physical, and often more “stereotypical,” violence, may reduce stigma and enhance disclosure of violence that otherwise may not be perceived as legitimate. The study also underscores the importance of social support across violence types, suggesting that it can be vital to ensure sufficient access to low-threshold services. Such services may provide emotional and practical support to individuals regardless of their informal social network. Although there seemed to be differences in how various violence characteristics and interpersonal factors were associated with disclosure of sexual and physical violence, similarities suggest that there might be shared pathways to disclosure that can enhance the well-being and recovery of individuals and societies impacted by interpersonal violence.

## Supplemental Material

sj-docx-2-jiv-10.1177_08862605261441617 – Supplemental material for Original ResearchDisclosure of Sexual and Physical Violence: The Role of Violence Characteristics, Social Support, and Trauma-Related Shame in a Norwegian Population-Based Sample Supplemental material, sj-docx-2-jiv-10.1177_08862605261441617 for Original ResearchDisclosure of Sexual and Physical Violence: The Role of Violence Characteristics, Social Support, and Trauma-Related Shame in a Norwegian Population-Based Sample by Louisa Cheng Seifert, Ida Frugård Strøm, Tore Wentzel-Larsen, Anja Duun Skauge and Maria Teresa Grønning Dale in Journal of Interpersonal Violence

sj-docx-3-jiv-10.1177_08862605261441617 – Supplemental material for Original ResearchDisclosure of Sexual and Physical Violence: The Role of Violence Characteristics, Social Support, and Trauma-Related Shame in a Norwegian Population-Based Sample Supplemental material, sj-docx-3-jiv-10.1177_08862605261441617 for Original ResearchDisclosure of Sexual and Physical Violence: The Role of Violence Characteristics, Social Support, and Trauma-Related Shame in a Norwegian Population-Based Sample by Louisa Cheng Seifert, Ida Frugård Strøm, Tore Wentzel-Larsen, Anja Duun Skauge and Maria Teresa Grønning Dale in Journal of Interpersonal Violence

sj-pdf-1-jiv-10.1177_08862605261441617 – Supplemental material for Original ResearchDisclosure of Sexual and Physical Violence: The Role of Violence Characteristics, Social Support, and Trauma-Related Shame in a Norwegian Population-Based Sample Supplemental material, sj-pdf-1-jiv-10.1177_08862605261441617 for Original ResearchDisclosure of Sexual and Physical Violence: The Role of Violence Characteristics, Social Support, and Trauma-Related Shame in a Norwegian Population-Based Sample by Louisa Cheng Seifert, Ida Frugård Strøm, Tore Wentzel-Larsen, Anja Duun Skauge and Maria Teresa Grønning Dale in Journal of Interpersonal Violence
