# Supplementary material for: Disclosure of Sexual and Physical Violence: The Role of Violence Characteristics, Social Support, and Trauma-Related Shame in a Norwegian Population-Based Sample
Source: J Interpers Violence. 2026 May 7;41(19-20):8188–218. doi: 10.1177/08862605261441617 (PMC13570981; doi:10.1177/08862605261441617)

Flow chart of Sampling Procedure

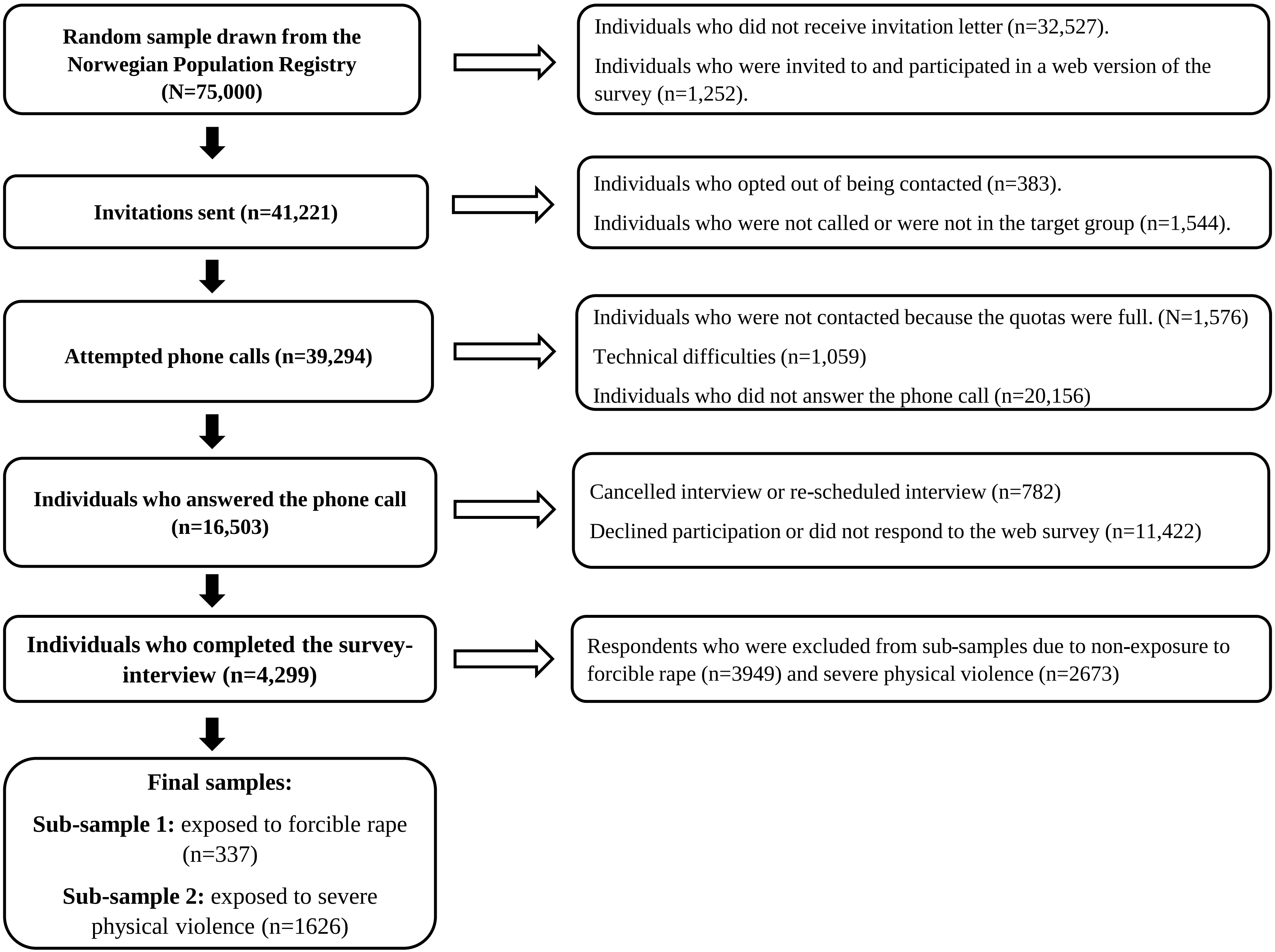

Supplement: Supplementary file 1 [file sj-pdf-1-jiv-10.1177_08862605261441617.pdf]
