# Supplementary material for: Disclosure of Sexual and Physical Violence: The Role of Violence Characteristics, Social Support, and Trauma-Related Shame in a Norwegian Population-Based Sample
Source: J Interpers Violence. 2026 May 7;41(19-20):8188–218. doi: 10.1177/08862605261441617 (PMC13570981; doi:10.1177/08862605261441617)
Supplement: Supplementary file 2 [file sj-docx-2-jiv-10.1177_08862605261441617.docx]

**Supplementary material**

**Table:** Bootstrapped differences in odds ratio estimates between sub-samples: individuals exposed to 1) forcible rape and 2) severe physical violence

|  | **Bootstrapped OR differences between sub-samples** | **95% CI lower** | **95% CI upper** |
| --- | --- | --- | --- |
| Differences in relationship to the perpetrator: known | 1.27 | 0.44 | 2.37 |
| Differences in relationship to the perpetrator: Stranger | 1.86 | 0.69 | 3.51 |
| Differences in perceived social support | -0.58 | -1.82 | 0.16 |
| Differences in trauma-related shame | -0.38 | -1.02 | 0.13 |
