# Supplementary material for: Disclosure of Sexual and Physical Violence: The Role of Violence Characteristics, Social Support, and Trauma-Related Shame in a Norwegian Population-Based Sample
Source: J Interpers Violence. 2026 May 7;41(19-20):8188–218. doi: 10.1177/08862605261441617 (PMC13570981; doi:10.1177/08862605261441617)
Supplement: Supplementary file 3 [file sj-docx-3-jiv-10.1177_08862605261441617.docx]

| **Variables** | **Mean (SD)** |
| --- | --- |
| **Trauma-related shame** | 0.19 (0.38) |
| **Perceived social support** | 3.29 (0.59) |
| ^1^ Individuals exposed to life-threatening illness; life-threatening accident; death of a close family member, partner, or very close friend in an accident, homicide, or suicide; bullying at primary or secondary school (intentionally teased or called names; spread rumors or gossip about; left out of a group or ignored; hit, someone stole or damaged personal belongings with, in a defenseless situation). Individuals with exposure to various forms of childhood and adulthood sexual or physical violence were excluded to serve as a comparison group. | |
|  | |

**Table:** Comparison group with exposure to potentially traumatic events^1^ excluding sexual and physical violence (n=283)
